# The Relationship Between Non-symbolic and Symbolic Numerosity Representations in Elementary School: The Role of Intelligence

**DOI:** 10.3389/fpsyg.2019.02724

**Published:** 2019-12-05

**Authors:** Tatiana Tikhomirova, Yulia Kuzmina, Irina Lysenkova, Sergey Malykh

**Affiliations:** ^1^Department of Psychology, Lomonosov Moscow State University, Moscow, Russia; ^2^Psychological Institute of Russian Academy of Education, Moscow, Russia; ^3^Department of Psychology, Kyrgyz-Russian Slavic University, Bishkek, Kyrgyzstan

**Keywords:** non-symbolic representation, approximate number system, symbolic representation, number line, fluid intelligence, longitudinal study

## Abstract

This study aimed to estimate the extent to which the development of symbolic numerosity representations relies on pre-existing non-symbolic numerosity representations that refer to the Approximate Number System. To achieve this aim, we estimated the longitudinal relationships between accuracy in the Number Line (NL) test and “blue–yellow dots” test across elementary school children. Data from a four-wave longitudinal study involving schoolchildren in grades 1–4 in Russia and Kyrgyzstan (*N* = 490, mean age 7.65 years in grade 1) were analyzed. We applied structural equation modeling and tested several competing models. The results revealed that at the start of schooling, the accuracy in the NL test predicted subsequent accuracy in the “blue–yellow dots” test, whereas subsequently, non-symbolic representation in grades 2 and 3 predicted subsequent symbolic representation. These results indicate that the effect of non-symbolic representation on symbolic representation emerges after a child masters the basics of symbolic number knowledge, such as counting in the range of twenty and simple arithmetic. We also examined the extent to which the relationships between non-symbolic and symbolic representations might be explained by fluid intelligence, which was measured by Raven’s Standard Progressive Matrices test. The results revealed that the effect of symbolic representation on non-symbolic representation was explained by fluid intelligence, whereas at the end of elementary school, non-symbolic representation predicted subsequent symbolic representation independently of fluid intelligence.

## Introduction

Considerable evidence suggests that the development of math competence is based on the ability to efficiently represent numerical magnitude information in symbolic formats and the acquisition of a symbolic number system (e.g., De Smedt et al., 2013; Schneider et al., 2017). The symbolic representation of numerosity is unique to humans and requires the ability to precisely represent numerosity verbally as number words or visually as Arabic number symbols (Dehaene and Cohen, 1995; Feigenson et al., 2004; De Smedt et al., 2013). The close link between symbolic numerosity representation and math achievement has been confirmed in several studies (e.g., Sasanguie et al., 2012; Rodic et al., 2015; Schneider et al., 2017). Therefore, it is important to understand how the symbolic representation of numerosity develops and how symbols acquire their numerical meanings. This question is usually referred to as “the symbolic grounding problem” (e.g., Leibovich and Ansari, 2016).

A widespread hypothesis posits that symbols acquire their meanings by being mapped onto pre-existed non-symbolic numerosity representations or an Approximate Number System (ANS). The ANS is usually defined as a system that allows individuals to perceive and approximately estimate numerosity without counting and using symbols (e.g., Feigenson et al., 2004; Dehaene, 2011). It has been postulated that the ability to represent and estimate numerosity in a symbolic format exists only in humans, whereas the ANS is evolutionarily ancient and innate. The ability to perceive numerosity in non-symbolic formats has been found in primates and non-human animals (Emmerton, 2001; Cantlon and Brannon, 2007; Agrillo et al., 2009). In humans, individual differences in this ability emerge early in childhood and exist even in infants (Lipton and Spelke, 2004; Xu and Arriaga, 2007). Moreover, it has been demonstrated that similar behavioral patterns can be found in animals, infants, children and adults. In particular, Cantlon and Brannon (2007) investigated non-symbolic arithmetic performance in monkeys and college students and found that the monkeys’ approximate mental arithmetic performance follows the same pattern as the students, who were tested using the same non-verbal addition task.

Several arguments support the idea that children acquire a symbolic number system by mapping these symbols onto approximate non-symbolic representations of numerosity (e.g., Mundy and Gilmore, 2009). First, it has been suggested that non-symbolic and symbolic magnitude representations adhere to the same behavioral patterns, which are known as numerical distance and size effects (e.g., Dehaene, 2001). It has been shown that compared to numerosities in both symbolic and non-symbolic formats, individuals are less precise and slower when comparing numbers or sets of objects that are more similar to each other or when there is a larger proportion between the numbers or sets of objects (numerical distance or numerical proportion effect) (e.g., Halberda and Feigenson, 2008; Holloway and Ansari, 2009). The size effect manifests as lower accuracy and a slower reaction time in comparing numbers and arrays of objects that are larger in size (Dehaene, 2001). The existence of the distance and size effects in symbolic and non-symbolic representation is usually explained by the overlapping of Gaussian curves reflecting the internal representation of each numerosity on a mental number line (e.g., Dehaene, 2003; Dietrich et al., 2015).

The second argument suggests that non-symbolic numerosity representation is associated with math achievement (e.g., Halberda et al., 2008; Libertus et al., 2013; Chen and Li, 2014; Keller and Libertus, 2015). In particular, it has been shown that non-symbolic arithmetic enables the acquisition of symbolic arithmetic skills (Gilmore et al., 2007). Hyde et al. (2014) showed that brief non-symbolic number practice enhances subsequent exact symbolic arithmetic in first graders. Some authors have demonstrated that the association between the ANS and symbolic math skills is stronger among children with low math performance than other children (Bonny and Lourenco, 2013; Purpura and Logan, 2015). This might indicate that the association between the ANS and symbolic skills is likely to be stronger when symbolic skills are at early stages of development.

Despite extensive evidence suggesting that the ANS may serve as the basis of the acquisition of symbolic numerosity representation and more complex math skills, some existing findings refute this hypothesis (for a review, Reynvoet and Sasanguie, 2016). First, whether the non-symbolic magnitude representation is associated with math achievement has been questioned. Some studies failed to find a significant effect of non-symbolic magnitude representation on math achievement (e.g., Inglis et al., 2011; Sasanguie et al., 2014). In many studies, the link between non-symbolic magnitude representation and math achievement became insignificant or dramatically decreased after controlling for any measures of symbolic magnitude representations or other cognitive abilities, such as inhibitory function (e.g., Lyons and Beilock, 2011; Gilmore et al., 2013; Kolkman et al., 2013; Sasanguie et al., 2013; Göbel et al., 2014).

The second argument against the ANS hypothesis of the “symbol grounding problem” is based on the results of studies demonstrating that non-symbolic and symbolic magnitude representations are distinct systems. In particular, it has been shown that the precisions of symbolic and non-symbolic representations are not significantly correlated and that both have an independent effect on math achievement at least in early school-aged children (Fazio et al., 2014; Guillaume et al., 2016; Matejko and Ansari, 2016; Sasanguie et al., 2017). It has also been shown that the symbolic and non-symbolic ratio effects are not correlated, suggesting that these two systems of numerosity representation are distinct (Lyons et al., 2015).

The third argument against the hypothesis that ANS serves a basis of the acquisition of the symbolic system is derived from several longitudinal studies that found that symbolic representations predicted subsequent non-symbolic representations rather than the opposite (Kucian et al., 2011; Mussolin et al., 2014; Shusterman et al., 2016). Specifically, it has been shown that at 3–4 years of age, children’s symbolic number knowledge predicts subsequent accuracy in non-symbolic magnitude comparisons, whereas the opposite link is not significant (Mussolin et al., 2014). Several studies have also shown that formal math education and experience with manipulating symbolic numbers enhance accuracy in non-symbolic comparisons (Kucian et al., 2011; Guillaume et al., 2013; Nys et al., 2013; Piazza et al., 2013). Thus, extensive evidence refutes the hypothesis that the acquisition of a symbolic number system occurs through the mapping of symbols onto ANS.

In addition, some authors argue that reciprocal relationships exist between ANS and symbolic representation systems (e.g., Toll et al., 2015; Goffin and Ansari, 2019). In particular, Toll et al. (2015) examined developmental changes in non-symbolic and symbolic comparison skills and demonstrated that there are bidirectional relationships. Goffin and Ansari (2019) proposed the possibility that the nature and direction of the associations between symbolic and non-symbolic numerosity representations may change depending on age and experience.

There are several possible issues in the studies concerning the relationship between ANS and symbolic magnitude representation that may hinder the generalization of the obtained results or result in contradictions in the findings. The first problem is related to issues with the measurement and operationalization of non-symbolic and symbolic representations. Notably, many studies confirmed that the ANS is the basis of the development of symbolic representations, used different symbolic math skills, such as number knowledge or arithmetic skills, and rarely used measurements of symbolic magnitude representations.

Even if symbolic representations were measured separately from more complex math skills, different tests might be used. The most popular measurements involve symbolic magnitude comparison tasks in which individuals compare two Arabic numbers and select the larger number (e.g., Toll et al., 2015; Matejko and Ansari, 2016), “give-a number task” (Mussolin et al., 2014; Shusterman et al., 2016) or Number Line task (NL) (e.g., Fazio et al., 2014). Although the results of different symbolic tests have high common dispersion (e.g., Laski and Siegler, 2007), it is possible that the relationship between non-symbolic and symbolic representations might vary due to differences in the measurement instruments.

The ability to represent numerosity in the non-symbolic format is mostly measured by various non-symbolic comparison tests in which individuals compare two arrays of objects (mostly dots) and determine which array is larger (e.g., Halberda et al., 2008; Libertus et al., 2011; Sasanguie et al., 2012; Smets et al., 2016). Several protocols of “dots” tests exist, such as the Panamath protocol (“blue–yellow dots test”) and the protocol described by Gebuis and Reynvoet (2011). The different types of “dots” tests may differ in their control of the visual parameters of the stimulus, which may seriously change the results of the ANS tests (e.g., Gebuis and Reynvoet, 2012; Szucs et al., 2013; Smets et al., 2016), and consequently, the power of the association between the ANS and symbolic math skills may also change.

The low consistency among the results of non-symbolic comparison tests, depending on the different ways used to control the visual parameters, questions the ability to process non-symbolic numerosity independently from perceptions of continuous visual properties, such as the cumulative area of two sets or a convex hull (e.g., Gebuis and Reynvoet, 2012; Clayton et al., 2015; Gilmore et al., 2016). Some authors have proposed that the development of precision in the ANS test is explained by an increase in the precision of the estimation of visual properties rather than specific numerosity perception (e.g., Leibovich and Henik, 2013; Gebuis et al., 2016). Other authors have suggested that although at an early age, accuracy in ANS tests is affected by the visual properties of a stimulus, the ability to estimate magnitude in non-symbolic format independently of the visual properties increases with age (Szucs et al., 2013; Tokita and Ishiguchi, 2013; Starr et al., 2017). However, the relationship between ANS and symbolic numerical skills might be partially explained by visuospatial skills.

The third problem is related to confounding variables. In longitudinal research, when developmental relationships between two constructs or variables are considered, it is important to consider other variables that could be correlated with both constructs. Some studies have demonstrated that both symbolic and non-symbolic skills are affected by executive function, intelligence or spatial ability (Xenidou-Dervou et al., 2015; Chew et al., 2016; Price and Wilkey, 2017). In various studies, non-verbal intelligence is significantly linked to a wide range of symbolic math skills, such as number line precision, arithmetic skills and number knowledge (e.g., Bachot et al., 2005; Geary et al., 2008; LeFevre et al., 2013; Östergren and Träff, 2013; Chu et al., 2016). Consequently, non-symbolic and symbolic representations might be correlated because they are affected by the same cognitive functions.

The fourth problem involves the methodology of the studies. Most studies investigating the association between ANS and symbolic magnitude representations were cross-sectional, which restricted their ability to draw conclusions regarding causality or even the direction of the effect. In cross-sectional studies, it is impossible to determine whether ANS serves as the foundation of symbolic representation or vice versa. To draw conclusions regarding the direction of the link, longitudinal studies are needed. Moreover, importantly, each variable should be measured at each time point to control for the previous level of the variables of interests (Goffin and Ansari, 2019).

In longitudinal studies performed to estimate developmental relationships between variables, it is possible to create different path models using the manifested scores of each variable (such as the proportion of correct answers). However, some studies have demonstrated that using manifested variables in path analyses might lead to biased estimations of the relationships between the variables (Coffman and MacCallum, 2005; Cole and Preacher, 2014). Instead of using manifested variables, it is recommended to apply the latent variable approach and structural equation modeling (Cole and Preacher, 2014). For example, in Wong et al. (2016) study, ANS was identified as a latent construct measured by non-symbolic comparison, non-symbolic addition, non-symbolic subtraction and non-symbolic multiplication. These authors also identified latent variable “Mapping,” which refers to symbolic number processing and was measured by numerosity naming, numerosity production and the NL test.

Unfortunately, longitudinal studies involving relatively large samples can rarely use several measures of one ability. When only one test of one ability is used, an alternative approach that might be used is parceling (aggregated estimation of several items) and the creation of latent variable with several parcels per construct (Little et al., 2002, 2013). Coffman and MacCallum (2005) demonstrated that using parcels and a specification of latent constructs with these parcels is better than using manifested variables in path analyses to obtain more reliable estimations of the associations between variables. Although some researchers have expressed concerns regarding the use of parcels, parceling offers some advantages in cases of the unidimensionality of the latent constructs (Little et al., 2013).

In summary, to estimate the extent to which ANS might serve as a basis for the development of symbolic numerosity representation and the acquisition of the numerical meaning of symbols, it is important to consider several aspects. It is important to use longitudinal designs and select relevant and reliable measures of symbolic and non-symbolic numerosity representations. It is also important to control for possible confounders, such as general cognitive abilities, as previous studies have demonstrated significant correlations with symbolic numerosity processing (e.g., Hornung et al., 2014; Namkung and Fuchs, 2016).

In this study, we aim to determine whether the development of symbolic representations occurs by mapping symbols on ANS. To fulfill this goal, we estimate the longitudinal relationship between non-symbolic comparison skills, which are related to the ANS, and precision in the NL test, which is related to symbolic representation, using a four-wave longitudinal study involving elementary schoolchildren.

The NL test was selected for several reasons. First, this test is widely used in studies concerning symbolic representations and their relationship with non-symbolic representations and math achievement. Precision in the NL test is consistently correlated with different types of math performances (De Smedt et al., 2009; Göbel et al., 2014; Friso-van den Bos et al., 2015; for a meta-analysis, see Schneider et al., 2018). Moreover, the correlation remained significant after controlling for domain-general (working memory and intelligence) and domain-specific (non-symbolic magnitude representation and proportional reasoning) abilities (Bailey et al., 2014; Hornung et al., 2014). Second, NL test results are highly correlated with another task reflecting symbolic magnitude representation, i.e., the number comparison task (e.g., Laski and Siegler, 2007). Third, NL test results are more highly correlated with math achievement than the symbolic magnitude comparison task (Schneider et al., 2018).

Although most authors agree that the NL test is a good instrument for measuring symbolic magnitude representations, some authors propose that the NL test measures number-numerosity mapping skills (e.g., Kolkman et al., 2013; Wong et al., 2016). Thus, NL test results might reflect both symbolic representations and mapping skills. From this point of view, the estimation of the developmental relationship between ANS and NL precision might shed on light on the “symbolic grounding problem.” If the acquisition of the meaning of symbols is based on mapping symbols onto pre-existed ANS, precision in the NL test should be affected by the precision of ANS.

To estimate the developmental relationship between precision in the NL test and ANS, we controlled for intelligence and estimated the extent to which the relationship between symbolic (or mapping) and non-symbolic skills might be explained by common dispersion with fluid intelligence. We hypothesize that if the relationship between symbolic and non-symbolic representations is attributed to the shared involvement of intelligence, the links between these constructs will become insignificant after controlling for FI. If the relationship between symbolic and non-symbolic representations is not explained by FI, the links between these constructs should remain significant after including intelligence in the model.

## Materials and Methods

### Participants

This study was conducted using data collected from 612 schoolchildren in grades 1–4 in Russia and Kyrgyzstan who participated in an ongoing longitudinal project named the “Cross-cultural Longitudinal Analysis of Student Success” (CLASS) project. One school was selected in both Russia and Kyrgyzstan. In both schools, the instruction was provided in Russian. The schools were equal in terms of rating within their region (e.g., the ratio of the average school scores on the final state mathematics examination to the average scores in the region), teacher characteristics (e.g., the ratio of teachers with higher pedagogical education to the total number of teachers and teachers’ experience and age) and curriculum (the mathematics and Russian language programs at the primary, secondary and high school levels were the same). The two samples did not differ in the family educational level. The proportion of mothers who had a higher education was 50.38% in the Russian sample and 52.65% in the Kyrgyz sample.

In both countries, all children studying in the first grade in the selected schools at the start of the longitudinal project participated in the study. The reasons for non-participation included illness or absence from school on the date of testing. We analyzed the patterns of missing data in the sample and confirmed the MCAR (missing completely at random) assumption by Little’s (1988) MCAR test. This test was insignificant (Chi-square distance = 272.51, df = 248, *p* = 0.14), indicating that MCAR assumption holds. Therefore, since the MCAR assumption holds and the sample size is sufficient, it was possible to apply listwise deletion to obtain adequate parameter estimates (Coertjens et al., 2017).

As at least three time points are necessary to carefully estimate developmental trajectories and development relationships (e.g., Duncan and Duncan, 2009; Curran et al., 2010), data from the schoolchildren who participated once or twice were removed from the analysis. The final sample consisted of 490 participants (51% girls); of these participants, 27% participated three times, and 73% participated four times. The mean age of the children at Time 1 was 7.61 years (*SD* = 0.40, range 6.42 – 8.83), at Time 2, the mean age was 8.58 (*SD* = 0.42, range 7.33 – 9.83), at Time 3, the mean age was 9.61 (*SD* = 0.43, range 8.33 – 10.83), and at Time 4, the mean age was 10.56 (*SD* = 0.41, range 9.33 – 10.75).

This study received approval from the Ethics Committee of the Psychological Institute of the Russian Academy of Education. Parental informed and written consent was obtained prior to the data collection. Consent was obtained from the children orally.

### Procedures and Materials

All participants were tested in quiet settings within their school facilities by a trained experimenter, and all measurement waves occurred at the end of the academic year (April–May). All experimenters strictly used the same protocol with instructions for the testing administration across all measurements. An experimenter with the help of two to three training adults monitored the execution of the tasks.

The experiment was performed in a computer classroom in groups of 14–15 pupils. Each participant sat in front of an individual monitor screen and performed the experiment independently. Each computer had a 17′′ LCD display with a resolution of 1,440 - 900 pixels and a refresh rate of 60 Hz. The participants were seated approximately 60 cm from the screen.

Each participant performed the “blue–yellow dots” test and NL test at each time point on a computer, and on the following 1–2 days, they performed the Raven’s Standard Progressive Matrices (SPM) test in paper-and-pencil format. The sequence of the tests was the same at each time point.

#### NL Test

This task was programed and adapted online from a description obtained from Siegler and Opfer (2003) (Tosto et al., 2013). A line was presented on the screen with a number at the top of the screen. An 11.5-pixel-high vertical mark indicated the start and end of the number line. The left end of the line was marked with a “0,” and the right end was marked with the number “1,000.” The total length of the line was 500 pixels, allowing the line to be correctly displayed on the computer screen. The center of the number line was at the center of the screen. The target number was 0.4 cm in height and placed 3 cm above the center of the number line.

The task required the participants to place the number displayed along the line. In total, 22 numbers were estimated, and these numbers were presented to all participants in the same order at various time points as follows: 246, 179, 818, 78, 722, 150, 366, 122, 738, 5, 147, 938, 18, 606, 2, 34, 754, 100, 56, 163, 486, and 725.

Each pupil could move the mouse to mark the position of the estimated number. The movement of the mouse coincided with the movement of a vertical red line (18.5 pixels) on the number line. When an individual decided to give an answer and mark the position, s/he clicked on the left mouse button.

There was only one practice in this test trial to reduce the effects of training as training has been shown to positively affect estimation accuracy. It was possible to take breaks. On each screen, there was an option to continue with the task or resume it later.

#### “Blue–Yellow Dots” Test

In this version of the “blue–yellow dots” test, the participants were presented with arrays of yellow and blue dots mixed together that varied in size and number. The task required the participants to judge whether the array contained more yellow or blue dots by pressing the corresponding keys on the keyboard. If an individual believed that the set contained more yellow dots, s/he pressed the “ж” key (corresponding to the “:” key on a QWERTY keyboard). If an individual believed that the set contained more blue dots, s/he pressed the “c” key (corresponding to the “c” key on a QWERTY keyboard).

The stimuli included 150 static pictures, and the arrays of yellow and blue dots were presented in intermixed format. The dot presentation varied between 5 and 21 dots of each color, and the ratios of the arrays of the two colors fell between 1:3 and 6:7. In each trial, the cumulative area of the set containing more dots was larger. The ratio of the cumulative areas of the two sets (the smallest area divided by the largest area) ranged between 0.30 to 0.99. In all trials, the average size of the yellow dots was equal to the average size of the blue dots.

The stimulus flashed on the screen for 400 ms, and the maximum response time was 8 s. If no answer was given during this time, the answer was recorded as incorrect, and a message appeared on the screen to encourage the participant to press the space bar to continue to the following trial. The message disappeared after 20 s, and the next stimulus was displayed only after pressing the space bar. The task included a set of instructions, a practice trial with two items and the option to repeat the practice. The presentation order was the same for all participants at each time point. It was possible to take breaks after each of the 50 trials.

#### Raven’s SPM Test

Raven’s SPM test is often used to measure fluid intelligence. The original version of the test comprises 5 sets, i.e., A, B, C, D, and E. Within each set, 12 items progressively become more difficult; thus, there were 60 tasks in total (Raven and Raven, 1998). There was no discontinuity rule, and all participants performed all tasks. The sum of correct answers in each block was calculated.

### Statistical Approach

The accuracy in the NL and “blue-yellow dots” tests can be calculated by using several approaches. For the NL test, several indicators of accuracy exist. The first indicator reflects the estimates of the deviation of the marked position of the numbers from the actual position of the number, which can be divided on a scale of estimates (e.g., Absolute Error Rates, Siegler and Booth, 2004) or used in the absolute term (e.g., Geary, 2011). The second indicator is the pattern of the estimates. For each individual, several models (e.g., logarithmic, exponential, and linear) of the relationship between the actual and marked numbers are estimated, and the fit indices of each model are calculated (proportion of explained variance). Then, the proportion of individuals whose estimates were the best fit by each model is calculated (e.g., Siegler and Opfer, 2003; Siegler and Booth, 2004). It has been demonstrated that the correlations between the NL test results and math achievement were higher using estimate deviations from the actual position than using the model fit indices (Schneider et al., 2018). Therefore, for the current analysis, we selected the deviation from the actual position as an indicator of NL precision. The higher the deviation, the lower the precision in the NL test.

The precision of ANS in the different dot test can be measured by the proportion or sum of correct answers (accuracy), reaction times, numerical distance effect or Weber fraction (*w*), which indicates the minimum proportion of two sets that can be detected by the participants. Thus, smaller Weber fractions indicate that an individual was able to differentiate numerosities that were more similar to each other. The proportion of correct answers and *w* were highly correlated in cross-sectional studies (e.g., Inglis and Gilmore, 2014; Dietrich et al., 2016; Tosto et al., 2017). It has also been demonstrated that among four possible indicators of precision in the dot test, proportion of correct answers had the highest test–retest reliability (Inglis and Gilmore, 2014). In the current study, we used the sum of correct answers as an indicator of accuracy in the “blue-yellow dots” test.

Since using the raw scores of the variables might lead to a biased estimation of the paths among the manifested variables (Cole and Preacher, 2014), we did not use the mean accuracy of both tests in the path analysis. Instead, we specified ANS and NL as latent constructs with three parcels per construct (Coffman and MacCallum, 2005) and used structural equation modeling to estimate the longitudinal relationship between the results of the NL test and the “blue-yellow dots” test across the children in grades 1–4.

For the NL test, each parcel was calculated as the mean deviation of the estimated number’s position from the actual position divided by 1,000 (as we had a “0–1,000” NL scale) for 7–8 sequential items. In summary, three parcels were created from 22 trials. The first parcel was calculated as the average deviation divided by 1,000 for the first seven numbers, the second parcel was calculated as the average deviation divided by 1,000 for the next seven numbers and the third parcel was calculated as the average deviation divided by 1,000 for the last eight numbers.

**FIGURE 1:**
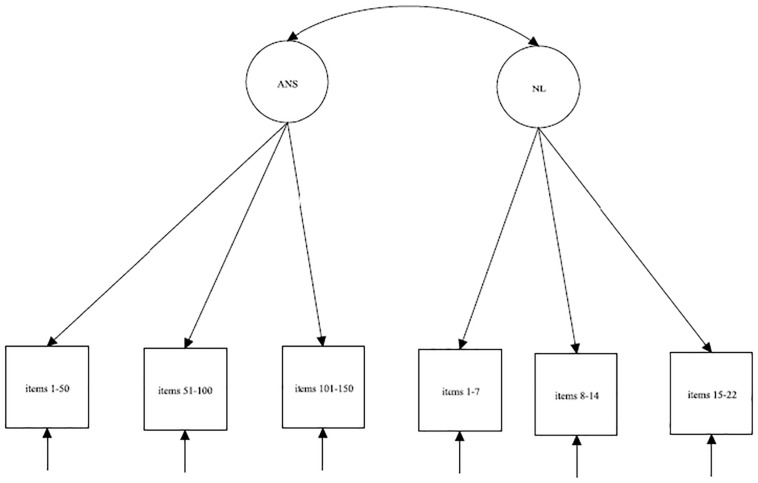
Schematic diagrams for the measurement models for ANS and NL.

For the ANS test, each parcel was calculated as the sum of correct answers among 50 sequential items. In summary, there were 150 trials in the ANS test, and the following three parcels were created: the first parcel was calculated as the sum of correct answers on items 1–50, the second parcel was calculated as the sum of correct answers on items 51–100 and the third parcel was calculated as the sum of correct answers on items 101–150. After calculating the parcels, we specified the measurement models for each grade separately (Figure 1).

**FIGURE 2:**
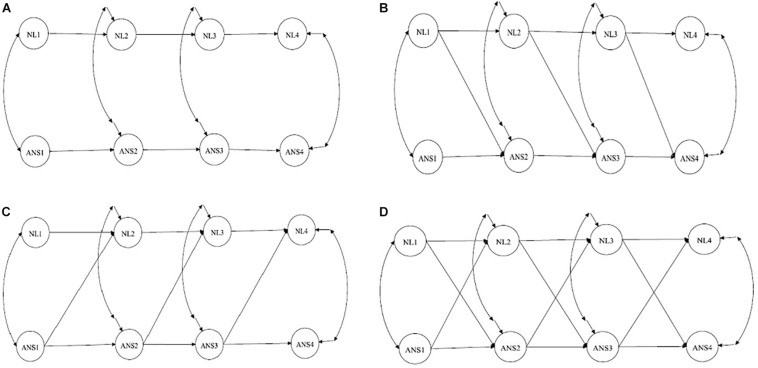
Schematic path diagrams for the four competitive models. **(A)** – Autoregressive model; **(B)** – NL affects ANS; **(C)** – ANS affects NL; **(D)** – ANS and NL have a reciprocal relation. ANS, approximate number sense; NL, number line.

After specifying the measurement models, we assessed and compared four competing structural models to select the best fitting model. The schematic path diagrams of each model are shown in Figure 2. The first model (Figure 2A) was an autoregressive model with no cross-lagged effects and only temporal stability and contemporary associations. This model implied that there were no developmental associations between symbolic and non-symbolic representations and that the two types of numerosity representations developed independently of each other.

In the second model (Figure 2B), the cross-lagged pathways from NL in the previous grade to ANS in the subsequent grade were added to test the hypothesis that NL had an effect on subsequent ANS while the ANS was not predictive of NL accuracy. This model implies that ANS was not the basis of the acquisition of symbolic representations and that, vice versa, symbolic skills may enhance ANS precision.

The third model (Figure 2C) included the cross-lagged pathways from ANS in the previous grade to NL in the subsequent grade to test the hypothesis that the precision of the NL estimation was predicted by the previous level of ANS accuracy. This model corresponds to the “ANS hypothesis” in the “symbolic grounded problem.” The final model represented reciprocal effects and tested the effects in both directions (Figure 2D). In each model, the correlations between ANS and NL within one grade were added. We also added autoregressive paths from ANS and NL in first grade to ANS and NL in third grade and from ANS and NL in second grade to ANS and NL in fourth grade, which significantly improved the model fit.

After selecting the best fitting model of the relationship between ANS and NL, we specified measurement models with the ANS, NL and FI latent constructs within each grade. FI was specified as a latent construct with five parcels. Each parcel was calculated as the sum of correct answers per block in Raven’s SPM test (A, B, C, D, and E). Then, we included FI in each grade as a predictor of subsequent ANS and NL to examine how the relationship between ANS and NL changed after controlling for FI. We included a country variable (0 = Kyrgyzstan, 1 = Russia) as a predictor to control for between-country differences in the latent constructs. In the following step, we tested a model in which FI had reciprocal relationships with ANS and NL.

The maximum likelihood estimator was applied. To compare the models, we used the chi-square difference test. The analysis was conducted with Mplus 7.0 software (Muthén and Muthén, 2012).

## Results

### Descriptive Statistics

The descriptive statistics of the parcels of each test and grade are presented in Table 1.

**TABLE 1:** Descriptive statistics for the total scores and parcels from first to fourth grades.

**Grade**	**Test**	**Mean**	***SD***	**Min**	**Max**
1	ANS (total scores)	92.20	14.13	61	124
	ANS1 (items 1–50)	32.37	5.99	16	45
	ANS2 (items 51–100)	29.92	5.38	17	45
	ANS3 (items 101–150)	29.90	5.46	18	46
	NL (total scores)	0.16	0.96	0.03	0.54
	NL1 (items 1–7)	0.16	0.09	0.02	0.49
	NL2 (items 8–14)	0.16	0.11	0.003	0.58
	NL3 (items 15–22)	0.16	0.10	0.015	0.67
	Raven’s SPM (total)	28.16	10.65	4	53
	Block A	9.28	1.84	1	12
	Block B	7.73	3.30	0	12
	Block C	5.26	3.05	0	11
	Block D	4.54	3.52	0	12
	Block E	1.34	1.95	0	12
2	ANS (total scores)	95.47	12.99	56	124
	ANS1 (items 1–50)	33.89	5.66	17	45
	ANS2 (items 51–100)	30.91	4.80	18	44
	ANS3 (items 101–150)	30.67	5.18	14	44
	NL (total scores)	0.13	0.08	0.02	0.45
	NL1 (items 1–7)	0.13	0.08	0.02	0.55
	NL2 (items 8–14)	0.12	0.10	0.01	0.66
	NL3 (items 15–22)	0.13	0.09	0.02	0.62
	Raven’s SPM (total)	33.88	9.66	3	54
	Block A	10.03	1.64	1	12
	Block B	8.76	2.75	0	12
	Block C	6.82	2.69	0	12
	Block D	6.38	3.14	0	12
	Block E	1.89	1.99	0	9
3	ANS (total scores)	98.80	13.40	63	130
	ANS1 (items 1–50)	35.57	5.55	17	47
	ANS2 (items 51–100)	31.93	5.13	19	44
	ANS3 (items 101–150)	31.29	5.35	18	46
	NL (total scores)	0.09	0.06	0.01	0.50
	NL1 (items 1–7)	0.09	0.06	0.01	0.46
	NL2 (items 8–14)	0.08	0.07	0.004	0.57
	NL3 (items 15–22)	0.09	0.07	0.01	0.54
	Raven’s SPM (total)	38.56	8.13	11	60
	Block A	10.67	1.36	4	12
	Block B	10.14	2.23	1	12
	Block C	7.92	2.35	0	12
	Block D	7.24	2.45	0	12
	Block E	2.59	2.38	0	12
4	ANS (total scores)	100.29	13.73	63	131
	ANS1 (items 1–50)	35.93	5.51	15	47
	ANS2 (items 51–100)	32.15	5.27	18	47
	ANS3 (items 101–150)	32.19	5.36	19	45
	NL (total scores)	0.07	0.05	0.02	0.33
	NL1 (items 1–7)	0.08	0.05	0.01	0.34
	NL2 (items 8–14)	06	0.05	0.02	0.34
	NL3 (items 15–22)	0.07	0.05	0.01	0.38
	Raven’s SPM (total)	41.66	7.49	12	57
	Block A	10.91	1.22	4	12
	Block B	10.59	1.91	0	12
	Block C	8.32	2.11	0	12
	Block D	8.28	2.41	0	12
	Block E	3.55	2.46	0	11

*SD, standard deviation; Min, minimum; Max, maximum.*

### Measurement Models

In each grade in which we tested the measurement model, ANS and NL were represented by three parcels. We also tested models with FI as the latent construct, which was presented by five parcels. The results revealed that all measurement models had good fit indices at each grade (Tables 2, 3).

**TABLE 2:** Fit indices of the measurement models for each grade with ANS and NL.

	**Grade 1**	**Grade 2**	**Grade 3**	**Grade 4**
	**(*n* = 423)**	**(*n* = 469)**	**(*n* = 478)**	**(*n* = 472)**
BIC	5783.36	6754.14	6769.49	6858.82
Sample-size adjusted BIC	5723.07	6693.84	6709.19	6798.51
χ2	23.576	10.78	13.91	6.64
df	8	8	8	8
RMSEA	0.068	0.027	0.039	0.000
90% CI	0.037 – 0.100	0.000 – 0.064	0.000 – 0.073	0.000 – 0.048
CFI	0.987	0.998	0.995	1.00
TLI	0.975	0.995	0.991	1.00
SRMR	0.041	0.021	0.016	0.017

*BIC, Bayesian information criterion; RMSEA, root mean square error of approximation; 90% CI, 90% confidence interval for RMSEA; CFI, comparative fit index; TLI, Tucker Lewis index; SRMR, standardized root mean square residual.*

**TABLE 3:** Fit indices of the measurement models for each grade with ANS, NL and FI.

	**Grade 1**	**Grade 2**	**Grade 3**	**Grade 4**
BIC	11278.89	12688.84	12961.83	12931.38
Sample-size Adjusted BIC	11164.64	12574.58	12847.57	12817.13
χ2	122.86	62.46	52.03	69.69
df	41	41	41	41
RMSEA	0.067	0.033	0.023	0.038
90% CI	0.053 – 0.081	0.014 – 0.049	0.000 – 0.041	0.022 – 0.054
CFI	0.96	0.99	0.99	0.99
TLI	0.95	0.99	0.99	0.98
SRMR	0.048	0.029	0.026	0.039

*BIC, Bayesian information criterion; RMSEA, root mean square error of approximation; 90% CI, 90% confidence interval for RMSEA; CFI, comparative fit index; TLI, Tucker Lewis index; SRMR, standardized root mean square residual.*

### Structural Equation Modeling

#### Relationship Between ANS and NL

The fit indices of each structural model of the relationship between ANS and NL are shown in Table 4.

**TABLE 4:** Fit indices for the structural models with ANS and NL.

	**Model 1**	**Model 2**	**Model 3**	**Model 4**	**Model 5a^&^**	**Model 5b^&⁣&^**
	**(Autoregressive)**	**(NL predicts ANS)**	**(ANS predicts NL)**	**(Full reciprocal)**	**(Restricted reciprocal1)**	**(Restricted reciprocal2)**
Sample-size Adjusted BIC	25312.47	25315.06	25301.19	25305.02	25299.50	25299.51
χ2	283.09	276.62	262.75	257.52	258.05	261.07
df	202	199	199	196	198	199
RMSEA	0.029	0.028	0.026	0.025	0.025	0.025
90% CI	0.020 – 0.036	0.020 – 0.036	0.016 – 0.034	0.016 – 0.033	0.015 – 0.033	0.016 – 0.033
CFI	0.99	0.99	0.99	0.99	0.99	0.99
TLI	0.98	0.98	0.98	0.98	0.98	0.98
SRMR	0.061	0.054	0.039	0.035	0.036	0.039
Δχ^2^ (Δ df)		6.47 (3) (vs. Model 1)	20.34^∗∗∗^ (3) (vs. Model 1)	5.23 (3) (vs. Model 3)	4.71^∗^ (1) (vs. Model 3)	3.03 (1) (vs. Model 5a)

**^&^*Model 5a – Model 3 with additional pathway from NL at grade 1 to ANS at grade 2. ^&⁣&^Model 5b – Model 5a without path from ANS at grade 1 to NL at grade 2. ^∗^*p* < 0.05, ^∗∗∗^*p* < 0.001.*

**FIGURE 3:**
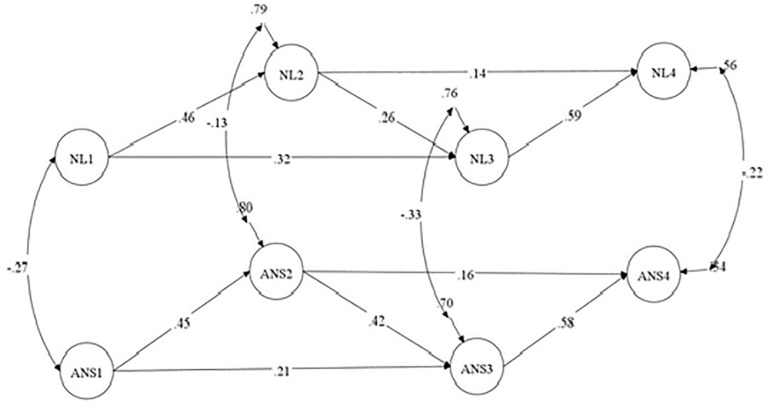
Autoregressive model. ANS, approximate number sense; NL, number line; Standardized coefficients are demonstrated; paths from the manifested variables (parcels) to the latent constructs are not shown.

The autoregressive model demonstrated a satisfactory fit to the data. The analysis revealed significant paths from ANS in grade 1 to ANS in grade 2 and grade 3, from ANS in grade 2 to ANS in grade 3 and grade 4, and from ANS in grade 3 to ANS in grade 4. These patterns of autoregressive paths were also obtained for NL (Figure 3).

Model 2 with the cross-lagged path from NL to ANS did not show a significant improvement in terms of fit to the data compared to the autoregressive model [Δχ^2^(3) = 6.47, *p* > 0.10]. Only one cross-lagged path, i.e., the path from NL in grade 1 to ANS in grade 2, was significant (β = −0.14, *SE* = 0.06, *p* < 0.05). The negative coefficient indicated that decreasing the deviation from the actual position of the number on the NL was correlated with increased accuracy in the non-symbolic comparison.

In contrast, Model 3 showed significant improvement over the autoregressive model after adding the cross-lagged paths from ANS to NL [Δχ^2^(3) = 20.34, *p* < 0.001]. ANS in grade 2 predicted NL in grade 3 (β = −0.16, *SE* = 0.05, *p* < 0.01], and ANS in grade 3 predicted NL in grade 4 (β = −0.13, *SE* = 0.05, *p* < 0.05). The path from ANS in grade 1 to NL in grade 2 was not significant.

**FIGURE 4:**
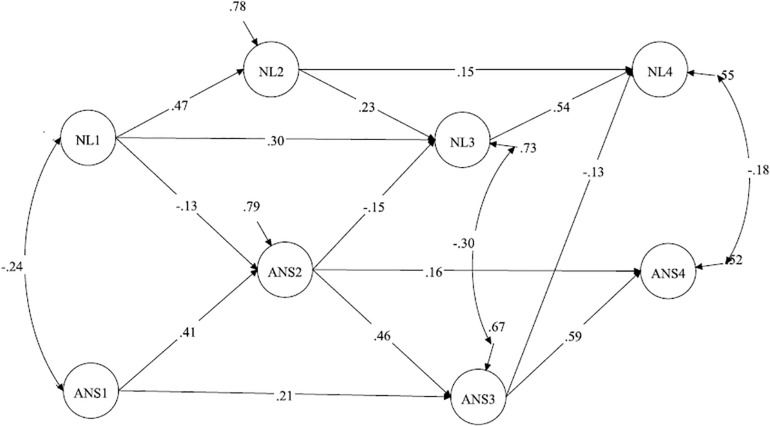
Model with restricted reciprocal relations between ANS and NL. ANS, approximate number sense; NL, number line; Standardized coefficients are demonstrated; paths from the manifested variables (parcels) to the latent constructs are not shown.

The full reciprocal model did not fit the data better than Model 3 [Δχ^2^(3) = 5.23, *p* > 0.10]. Next, we tested a restricted reciprocal model (Model 5a) in which the cross-lagged path from NL at grade 1 to ANS at grade 2 was added to the cross-lagged paths from ANS to NL. This model fit the data better than Model 3 [Δχ^2^(1) = 4.71, *p* < 0.05]. However, the path from ANS at grade 1 to NL at grade 2 was insignificant in Model 5a, and we thus tested Model 5b in which this path was excluded. A comparison of Models 5a and 5b demonstrates that Model 5b did not fit the data worse than Model 5a. Therefore, we selected Model 5b as a better fitting model. Therefore, the effects from NL to ANS were supported in grade 1, whereas ANS in grades 2 and 3 predicted subsequent NL (Figure 4).

#### Relationships Among ANS, NL, and FI

To test if the relationship between NL and ANS might be explained by common dispersion with FI, we added FI at each grade as a predictor of ANS and NL in the subsequent grades to the restricted reciprocal model as it was previously selected as a better fitting model (Model 6). We also added the country variable (Russia = 1) as a predictor of ANS, NL, and FI. The goodness of fit indices for this model are demonstrated in Table 5.

**TABLE 5:** Fit indices for the structural models with ANS, NL, and FI.

	**Model 6 (FI predicted ANS and NL, country differences)**	**Model 7 (reciprocal relationships between FI and ANS, NL)**
Sample-size Adjusted BIC	47968.45	47962.69
χ2	1304.89	1281.56
dF	833	827
RMSEA	0.034	0.033
90% CI	0.030 – 0.037	0.030 – 0.037
CFI	0.95	0.96
TLI	0.94	0.95
SRMR	0.049	0.044
Δχ^2^(Δ df)		23.33^∗∗∗^ (6) (vs. Model 6)

*^∗∗∗^*p* < 0.001.*

**FIGURE 5:**
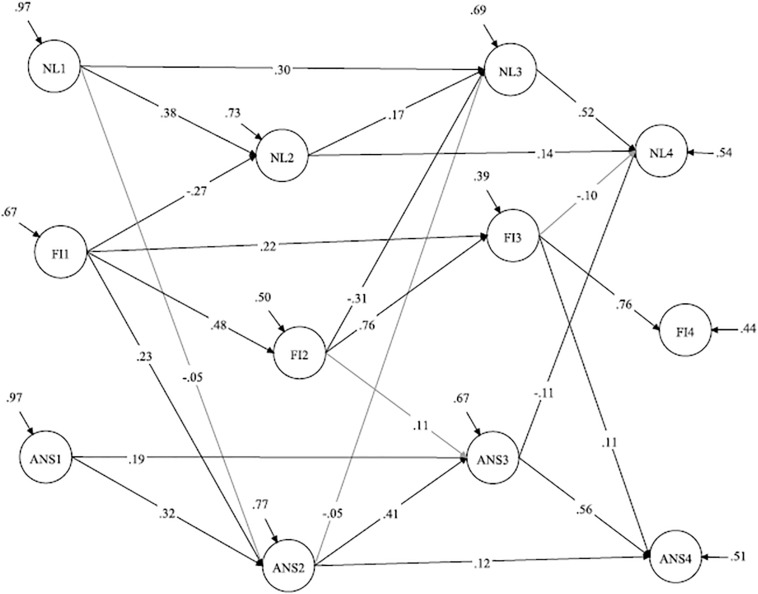
Model with restricted reciprocal relations between ANS and NL; FI is a predictor of ANS and NL (with country controlled). ANS, approximate number sense; NL, number line; FI, fluid intelligence; Standardized coefficients are demonstrated; paths from the manifested variables (parcels) to the latent constructs are not shown. Correlations between the constructs within one wave are not shown. The country variable is included in the model but is not shown. Insignificant paths are shown in gray.

The results of Model 6 reveal that FI in grade 1 predicted subsequent ANS and NL, whereas FI in grade 2 predicted NL in grade 3, and FI in grade 3 predicted ANS in grade 4. The paths from NL in grade 1 to ANS in grade 2 and from ANS in grade 2 to NL in grade 3 became insignificant, while the path from ANS in grade 3 to NL in grade 4 remained significant. Thus, NL in grade 3 was predicted by FI but not by ANS, whereas NL in grade 4 was predicted by ANS in grade 3 but not FI (Figure 5). Accordingly, FI eliminated the cross-lagged paths from grades 1 to 2 and from grades 2 to 3 but not from grades 3 to 4.

The cross-country differences were significant in grade 1 for ANS, NL and FI and in grade 2 for FI only; in grade 3, significant between-country differences existed in NL and FI, and in grade 4, there were no significant cross-country differences.

Then, we tested a model in which FI had reciprocal relationships with ANS and NL (Model 7). The goodness of fit indices of this model are presented in Table 5. A comparison of Models 6 and 7 reveals that Model 7 fit the data better than Model 6 [Δχ^2^ = 23.33 (6), *p* < 0.001].

**FIGURE 6:**
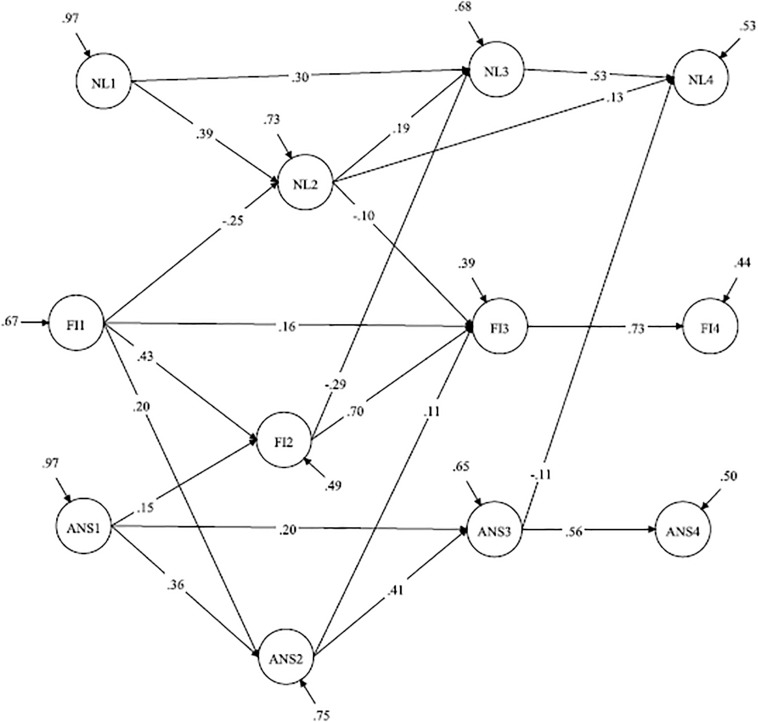
Model with restricted reciprocal relations between ANS, NL, and FI (with country controlled). ANS, approximate number sense; NL, number line; FI, fluid intelligence; Standardized coefficients are demonstrated; paths from the manifested variables (parcels) to the latent constructs are not shown. Only significant paths are shown. Correlations between the constructs within one wave are not shown. The country variable is included in the model but is not shown.

The results (see Table 6) reveal that FI in grade 1 predicted NL and ANS in grade 2, and FI in grade 2 predicted NL in grade 3. The path from FI in grade 3 to ANS in grade 4 became insignificant in Model 7; therefore, FI in grade 3 did not predict ANS or NL in grade 4. FI was also predicted by ANS or NL at different time points. In particular, FI in grade 2 was predicted by ANS in grade 1, and FI in grade 3 was predicted by ANS and NL in grade 2. FI in grade 4 was not predicted by the previous ANS or NL (Figure 6). Accordingly, the results reveal that FI had a reciprocal relationship between ANS or NL in grades 1–2, whereas the relationships between ANS and NL in these years were not significant when controlling for FI. Later, in grade 3, the effect of FI on subsequent ANS or NL became insignificant, whereas the effect of ANS in grade 3 to NL in grade 4 was significant.

**TABLE 6:** Standardized regression coefficients from the Model 7.

**Paths**	**ANS, NL and FI**	
	**B (*SE*)**	**95% CI**
**Autoregressive paths**		
ANS2 ON ANS1	0.36^∗∗∗^(0.06)	[0.24;0.49]
ANS3 ON ANS2	0.41^∗⁣∗∗^(0.06)	[0.29;0.54]
ANS3 ON ANS1	0.20^∗∗^(0.07)	[0.07;0.33]
ANS4 ON ANS2	0.12 (0.07)	[−0.01;0.26]
ANS4 ON ANS3	0.57^∗⁣∗∗^(0.06)	[0.45;0.68]
NL2 ON NL1	0.39^∗⁣∗∗^(0.05)	[0.29;0.49]
NL3 ON NL2	0.19^∗∗^(0.05)	[0.08;0.29]
NL3 ON NL1	0.30^∗⁣∗∗^(0.05)	[0.20;0.40]
NL4 ON NL2	0.13^∗∗^(0.05)	[0.04;0.23]
NL4 ON NL3	0.53^∗⁣∗∗^(0.05)	[0.43;0.62]
FI2 ON FI1	0.43^∗⁣∗∗^(0.06)	[0.31;0.54]
FI3 ON FI2	0.70^∗⁣∗∗^(0.06)	[0.58;0.82]
FI3 ON FI1	0.16^∗^(0.07)	[0.03;0.29]
FI4 ON FI3	0.73^∗⁣∗∗^(0.04)	[0.65;0.82]
**Cross-lagged paths**		
ANS2 ON NL1	−0.06(0.06)	[−0.17;0.06]
ANS2 ON FI1	0.20^∗∗^(0.08)	[0.05;0.35]
ANS3 ON FI2	0.09 (0.07)	[−0.05;0.23]
ANS4 ON FI3	0.10 (0.06)	[−0.01;0.21]
NL2 ON FI1	−0.25^∗⁣∗∗^(0.07)	[−0.38;−0.12]
NL3 ON ANS2	−0.07(0.06)	[−0.17;0.05]
NL3 ON FI2	−0.29^∗⁣∗∗^(0.06)	[−0.45;−0.17]
NL4 ON ANS3	−0.11^∗^(0.05)	[−0.21;−0.01]
NL4 ON FI3	−0.09(0.05)	[−0.19;0.01]
FI2 ON ANS1	0.15^∗∗^(0.05)	[0.05;0.25]
FI2 ON NL1	−0.02(0.05)	[−0.11;0.08]
FI3 ON ANS2	0.11^∗^(0.05)	[0.01;0.23]
FI3 ON NL2	−0.10^∗^(0.04)	[−0.19;−0.01]
FI4 ON ANS3	−0.01(0.06)	[−0.12;0.10]
FI4 ON NL3	−0.08(0.05)	[−0.18;0.02]
**Correlations within waves**		
ANS1 WITH NL1	−0.21^∗⁣∗∗^(0.06)	[−0.32;−0.10]
NL1 WITH FI1	−0.28^∗⁣∗∗^(0.05)	[−0.39;−0.17]
ANS1 WITH FI1	0.25^∗⁣∗∗^(0.06)	[0.13;0.37]
ANS2 WITH NL2	−0.05(0.06)	[−0.17;0.07]
NL2 WITH FI2	−0.12^∗^(0.06)	[−0.24;−0.004]
ANS2 WITH FI2	0.22^∗∗^(0.07)	[0.09;0.35]
ANS3 WITH NL3	−0.29^∗⁣∗∗^(0.06)	[−0.40;−0.17]
NL3 WITH FI3	−0.15^∗^(0.06)	[−0.27;−0.02]
ANS3 WITH FI3	0.09 (0.07)	[−0.05;0.23]
ANS4 WITH NL4	−0.18^∗^(0.07)	[−0.31;−0.04]
NL4 WITH FI4	−0.13(0.07)	[−0.27;0.003]
ANS4 WITH FI4	0.11 (0.08)	[−0.04;0.27]
**Country differences**		
NL1 ON country	−0.18^∗⁣∗∗^(0.05)	[−0.28;−0.09]
NL2 ON country	0.02 (0.06)	[−0.09;0.13]
NL3 ON country	0.14^∗^(0.05)	[0.04;0.25]
NL4 ON country	0.03 (0.04)	[−0.06;0.11]
ANS1 ON country	0.17^∗∗^(0.06)	[0.06;0.28]
ANS2 ON country	0.04 (0.07)	[−0.09;0.16]
ANS3 ON country	0.05 (0.06)	[−0.07;0.16]
ANS4 ON country	0.06 (0.05)	[−0.03;0.15]
FI1 ON country	0.57^∗⁣∗∗^(0.04)	[0.50;0.65]
FI2 ON country	0.31^∗⁣∗∗^(0.05)	[0.21;0.41]
FI3 ON country	−0.26^∗⁣∗∗^(0.05)	[−0.36;−0.16]
FI4 ON country	−0.05(0.04)	[−0.13;0.04]

*^∗∗∗^*p* < 0.001, ^∗∗^*p* < 0.01, ^∗^*p* < 0.05.*

## Discussion

The “symbolic grounded problem,” which can be defined as the question of how symbolic number systems develop and how symbols acquire their meanings, has been extensively discussed. A widely supported hypothesis posits that symbols acquire their meanings by mapping onto an innate and evolutionary ancient system of non-symbolic numerosity representations (ANS). Although some evidence confirms this hypothesis (e.g., Gilmore et al., 2007), several existing arguments contradict the “ANS hypothesis.” In particular, some studies have found no close relationship between symbolic numerosity representations and ANS at least at a young age (Matejko and Ansari, 2016), while other studies have demonstrated that in contrast to the “ANS hypothesis,” the acquisition of symbolic math skills may improve accuracy in non-symbolic representation, while the opposite links were non-significant (Mussolin et al., 2014).

It has been suggested that some contradictions might be explained by methodological issues regarding the measurement of both symbolic and non-symbolic representations, such as the use of different types of tasks, different measures of accuracy or different formats of stimulus presentation in tasks involving non-symbolic representations. Other existing issues are related to the lack of longitudinal studies and the problem of confounding variables in the estimation of developmental relationships between non-symbolic and symbolic representations.

Considering these issues, we aimed to estimate the developmental relationship between non-symbolic and symbolic representations using a four-wave longitudinal study involving schoolchildren from grade 1 to grade 4. Symbolic representations were measured by an NL test, and non-symbolic representations were measured by a “blue–yellow dots” test. We used the mean deviation from the actual position of the number as an indicator of accuracy in the NL test and the sum of the correct answers as an indicator of accuracy in the “blue–yellow dots” test. We specified ANS and NL as latent constructs, and each construct was presented by three parcels to avoid biased estimations of paths using a path analysis with manifested variables (Cole and Preacher, 2014).

Our analysis revealed that the restricted reciprocal model in which symbolic representation in grade 1 predicted non-symbolic representation in grade 2 and non-symbolic representation in grades 2 and 3 predicted subsequent symbolic representation fit the data significantly better than the other models. These results are consistent with some hypotheses regarding the bidirectional relationship between symbolic and non-symbolic representations (Goffin and Ansari, 2019).

Our results confirmed the “ANS hypothesis” but only at the end of elementary school. At the start of schooling, when children must rapidly acquire system number knowledge, symbolic representation is not predicted by non-symbolic representation and vice versa; accurate symbolic representations may lead to improvement in precision in ANS. These results are consistent with a study conducted by Mussolin et al. (2014), who demonstrated that at 3–4 years of age, children’s symbolic number skills predicted subsequent accuracy in non-symbolic magnitude comparisons, whereas the opposite links were non-significant. The children in our sample were older than those in the study conducted by Mussolin et al. (2014), but it is possible that this relationship pattern is typical during the period at the beginning of formal schooling when children acquire symbolic number knowledge.

It may also be the case that the acquisition of symbolic number systems during schooling can enhance the adjustment of the ANS through feedback. It has been demonstrated that feedback during the execution of non-symbolic number comparison tasks is associated with increased accuracy (DeWind and Brannon, 2012). Thus, the acquisition of symbolic number knowledge and counting provide the opportunity to compare the results of approximate and exact estimations of numerosity and tune the results of rapid and approximate estimation according to more exact symbolic representations.

These results are partially consistent with an alternative hypothesis of the “symbolic grounding problem,” which posits that an understanding of symbolic number systems is acquired not through mapping onto the ANS but through the association of numbers to an Object Tracking System (OTS), which refers to a system representing numbers in a very precise way but with limited capacity (3–4 items) (Reynvoet and Sasanguie, 2016). According to this hypothesis, an understanding of larger numerosity occurs through order association with smaller numbers rather than through the ANS. From this point of view, the growth in precision in NL should not be associated with the ANS.

Meanwhile, our study demonstrated that later, in grades 3–4, precision in the NL estimation was predicted by the ANS, which confirms the “ANS hypothesis” of the “symbolic grounding problem” but in a slightly different way.

Most likely, the effect of non-symbolic representation on symbolic representation emerges after a child masters the basics of symbolic number knowledge, such as counting in the range of 20 and simple arithmetic. According to the national educational standards in Russia and Kyrgyzstan, in the first grade, pupils should understand numbers from 0 to 20. During this period, the precision of the ANS does not predict NL accuracy because pupils may identify the position of a number on a number line by connecting larger numbers to smaller numbers. Subsequently, after students master more complex number knowledge (from 20 to 100 and from 100 to 1,000), ANS can serve as a basis for symbolic representation. It is possible that for relatively small numbers (0–10), the acquisition of the semantic meaning of symbols occurs through mapping to the OTS, while for relatively large numbers, such acquisition relies on mapping to the ANS. Future studies are needed to test this hypothesis.

The association between ANS and NL might be explained by the fact that they both represent numerosity processing. In addition, the NL test and “blue–yellow dots” test may be correlated because they both involve visuospatial skills. Specifically, it has been demonstrated that visuospatial skills, such as visuospatial working memory and mental rotation, are significantly correlated with NL precision (Geary et al., 2008; LeFevre et al., 2013; Simms et al., 2016). The accuracy of non-symbolic magnitude comparison is also affected by the visual properties of the stimulus (e.g., Gebuis and Reynvoet, 2012; Gilmore et al., 2013). Particularly, children can rely on a comparison of the total surface area between two compared sets of objects to make comparison judgments in the case of congruency between numerosity and visual cues (e.g., Gilmore et al., 2013; Starr et al., 2017). Consequently, pupils who are more precise in their estimation of visual cues in the non-symbolic comparison task might be more accurate in identifying the position of the number on the NL.

In this study, we could not identify the extent to which the relationship between accuracy in the “blue-yellow dots” test and NL test was explained by the involvement of visuospatial skills. However, notably, the involvement of visuospatial skills might explain the association between the two constructs but not the direction of this association. Thus, even if we control for visuospatial skills to explain the relationship between accuracy in the NL test and “blue-yellow dots” test, we could not explain why accuracy in the “blue-yellow dots” task predicted subsequent accuracy in the NL and vice versa.

To some extent, we control for visuospatial skills by including the accuracy in Raven’s SPM test. Numerous studies have demonstrated that Raven’s SPM test measures not only the g factor but also other factors, such as visualization and perceptual and spatial factors (e.g., van der Ven and Ellis, 2000; Lynn et al., 2004; Schweizer et al., 2007; Gignac, 2015). In particular, Lynn et al. (2004) identified the following three factors that are measured by Raven’s SPM test: gestalt continuation, visuospatial ability and verbal-analytic reasoning. Schweizer et al. (2007) contended that there is a correlation between performance on Raven’s SPM test and perceptual efficiency. Our results indicated that the paths from the NL test in grade 1 to ANS in grade 2 and from ANS in grade 2 to NL in grade 3 became insignificant after including accuracy in Raven’s SPM test in the model. However, the path from ANS in grade 3 to NL in grade 4 remained significant. This finding indicates that non-symbolic representation has an independent effect on the accuracy of symbolic representation, but this effect occurs later at the end of elementary school. We propose that at the beginning of schooling, symbolic and non-symbolic representations are related to each other because both require shared cognitive mechanisms, while in the process of education, these representations become more distinct from domain-general resources.

Furthermore, as the results of Model 7 demonstrate, in grades 1 and 2, FI predicts both subsequent symbolic and non-symbolic representations or separately symbolic representations. Later, in grade 3, FI does not predict non-symbolic or symbolic representations in grade 4. NL accuracy in grade 4 was predicted only by ANS and not FI. This finding can also indicate the growing independence of numerosity representations from more general cognitive abilities.

Although the estimation of cross-country differences was not an aim of our study, notably, there were significant cross-country differences in non-symbolic and symbolic representations in grade 1. These differences eventually became insignificant. In grades 1 and 2, FI was higher in the children from Russia, and in grade 3, the children from Kyrgyzstan demonstrated a higher accuracy in FI. In grade 4, there were no differences in FI between the Russian and Kyrgyz children. The differences in non-symbolic and symbolic representations may likely be due to different experiences with formal education before the beginning of school. Most children from the Russian sample (95%) attended kindergarten before school and were taught the number system and simple arithmetic. In contrast, the children from the Kyrgyz sample were less likely to have attended kindergarten (32%) and had less experience with formal education before school. In such cases, the Russian children had an advantage at the beginning of schooling, but this advantage disappeared from grade 1 to grade 4. However, future studies are needed to obtain a deeper understanding of the cross-country difference and its dynamic in FI.

Our study has several limitations. First, in our study, we used the version of the “blue-yellow dots” test in which all trials were congruent, as numerosity was positively correlated with the surface areas. The congruency of trials may impugn the validity of this test for the measurement of non-symbolic representations *per se*. Partly, this limitation may be overcome by using an intermixed format of stimulus presentation. It has been demonstrated that the reliability of this test is higher in an intermixed format than in the paired or sequential formats (Price et al., 2012). It has been also shown that the associations between mathematical achievement and accuracy in congruent and incongruent trials were exclusively significant in the intermixed task but not in a separate format of stimulus presentation (Norris and Castronovo, 2016). Therefore, we propose that the “blue–yellow dots” test is more sensitive to the measurement of non-symbolic representations in the intermixed format than in the separate format of presentations.

The second limitation refers to the age and educational experience of the participants. To investigate if ANS serves as a basis for the acquisition of symbolic number knowledge, it is necessary to start testing participants before they begin any formal education. In the current longitudinal project, we started testing pupils at the end of grade 1 when they had almost a full year of schooling. Therefore, the association between ANS and symbolic representation that was found in our study referred to a period when pupils already had number system knowledge to some extent. However, we assume that the acquisition of a symbolic number system does not limit the acquiring of numbers from 1 to 10 or to 20. The acquisition of a number system continues through all stage of formal education. Accordingly, our findings may shed on light on the developmental relations between ANS and symbolic representation in the period of elementary school when pupils may master some basis of a symbolic number system. The association between ANS and symbolic representation may change in different studies of education.

Therefore, we propose that non-symbolic representation has an effect on symbolic representation at the end of elementary school that is independent of fluid intelligence or visuospatial skills, whereas the effect of symbolic representation on the precision of non-symbolic comparison in the previous stage of formal education is explained by fluid intelligence or visuospatial skills. Future research is necessary to estimate the possible changes in the relationship among FI and symbolic and non-symbolic representations in secondary or high school. The ongoing longitudinal project CLASS will obtain results for further investigation of the development of and interrelations among these constructs.

## Data Availability Statement

The datasets generated for this study are available on request to the corresponding author.

## Ethics Statement

This study received approval from the Ethics Committee of the Psychological Institute of the Russian Academy of Education. Parental informed and written consent was obtained prior to the data collection. Consent was obtained from the children orally.

## Author Contributions

SM directs and received funding for the “Cross-cultural Longitudinal Analysis of Student Success” (CLASS) project. SM and TT conceived and designed the present study. YK and IL conducted the analyses and interpreted the results under the supervision of TT and SM. YK drafted the manuscript. All authors discussed the results and implications, commented on the manuscript at all stages, and approved the final version of the manuscript for submission.

## Conflict of Interest

The authors declare that the research was conducted in the absence of any commercial or financial relationships that could be construed as a potential conflict of interest.
